# Communication of Syphilis by Vaccine Lymph

**Published:** 1856-04

**Authors:** 


*Communication of Syphilis by Vaccine Lymph.*

The possibility of communicating syphilis by means of the vaccine lymph is utterly denied by Ricord and Cultierier of France, and Heyfelder and Pauli, two distinguished medical men of Rhenish Bavaria. This opinion we think will not be very readily adopted by the profession without the most indubitable proof, certainly not upon the mere *say so* of any men, however reputable and learned. The above opinion has been called out on the condemnation and imprisonment of a medical man in Bavaria, for vaccinating from a child having syphilitic eruptions.

